# Digital soft power of cultural heritage: domestic netizens' subjective perception and characteristics of the global influence of traditional festivals

**DOI:** 10.3389/fpsyg.2026.1896944

**Published:** 2026-07-22

**Authors:** Hua Tian

**Affiliations:** School of Marxism, Beijing Jiaotong University, Beijing, China

**Keywords:** collective self-esteem, cultural identity, cyberpsychology, digital contact and interaction level, digital cultural empowerment, media psychology, perceived global influence, traditional festivals

## Abstract

**Introduction:**

In the era of digital interconnection, cultural soft power extends beyond macro-policy communication, increasingly manifesting in the subjective perceptions and group identity evaluations formed by netizens during daily online interactions. This study explores the psychological mechanisms linking domestic netizens' digital engagement with their perceived global influence of Chinese traditional festivals, collective self-esteem, and sense of digital cultural empowerment.

**Methods:**

Using a cross-sectional survey (*N* = 1,540), we measured digital interaction levels, audiovisual modality dependency, and core cultural-psychological indicators. Data were statistically analyzed via confirmatory factor analysis, multi-group comparisons, and the PROCESS macro.

**Results:**

Results indicated that digital engagement was significantly and positively associated with perceived global influence. Moreover, perceived global influence significantly mediated the relationship between digital interaction and collective self-esteem. The data also supported a serial indirect path from digital interaction to digital cultural empowerment, sequentially mediated by perceived global influence and collective self-esteem. Notably, neither audiovisual modality dependency nor participants' overseas experience significantly moderated the core association between digital engagement and perceived global influence.

**Discussion:**

The findings support a statistical association consistent with the “cognition-emotion-empowerment” serial framework, suggesting that cultural identity formation and psychological empowerment in the digital age are more closely associated with the active frequency of everyday online interaction than with specific media formats (e.g., short videos vs. text) or direct intercultural experiences. Highlighting the characteristics of “mediated migration,” this study provides empirical, user-centric insights for optimizing the global digital communication strategies of traditional cultural heritage. Finally, because this study relies on cross-sectional survey data, all highlighted paths represent statistical associations rather than strict causal mechanisms.

## 1 Introduction

The ubiquitous integration of digital media into everyday life has fundamentally reshaped the ways cultural heritage is disseminated. Chinese traditional festivals, represented by the Spring Festival, Dragon Boat Festival, and Mid-Autumn Festival, originally relied primarily on family rituals, local customs, public celebrations, and intergenerational transmission for continuity. In the era of social media, user activities such as viewing, commenting on, sharing, and re-disseminating cultural content have become important forms of digital interaction (Aichner et al., 2021). These festivals are also continuously transformed into text-and-image posts, short video clips, live streaming scenes, hashtags, and comment interactions. Consequently, traditional festivals have become not only time nodes and ritual practices in real life but also cultural symbols that can be viewed, discussed, evaluated, and re-disseminated in cyberspace. In recent years, the communication value of intangible cultural heritage in tourism development, staged performances, and urban brand construction has received sustained attention, providing an important research background for understanding the re-dissemination of traditional festivals in the digital age (Chen et al., 2022; Demgenski, 2023; Zhang et al., 2024). Particularly, recent studies applying big data perspectives have highlighted the transformative role of digital platforms in evaluating the cultural heritage value of Chinese festivals (Li, 2024). Furthermore, investigating user engagement with digital Chinese cultural heritage underscores that online spaces not only connect information but also facilitate deep emotional sharing among participants (Tong et al., 2023).

Existing research on cultural soft power has mostly unfolded at macro levels such as national image, international communication, public diplomacy, and intercultural exchange. Soft power theory proposed by Nye emphasizes the importance of cultural attraction, value identity, and institutional image in international relations (Nye, 1990). Subsequent national image research further points out that a nation's cultural image is not a single dimension but is composed of multiple dimensions: functional, normative, aesthetic, and emotional (Buhmann and Ingenhoff, 2015). Meanwhile, spontaneous public experiences, active sharing, and network diplomatic interactions on digital platforms have gradually become important perspectives for measuring and understanding soft power (Shahin and Huang, 2024; Yeo et al., 2024). This perspective is fundamental to understanding cultural communication at the national level, but in the current digital media environment, the manifestation of cultural soft power does not exist only in policy texts, official communications, or international cultural projects. Ordinary netizens participate in the flow of cultural information at a daily level by browsing, liking, commenting, forwarding, and co-creating content around traditional festivals on social platforms. Relevant studies indicate that social media is not only a channel for information dissemination but may also participate in the process of preservation, expression, and re-confirmation of cultural identity among youth groups (Ghorzang, 2025). Digital and social media research also shows that platform interaction, user-generated content, and online communities are continuously changing individuals' information contact, attitude formation, and participation methods (Dwivedi et al., 2021). Indeed, user-generated content and digital footprints on social media have emerged as critical participatory tools for exploring and verifying cultural landscapes and public engagement (Li et al., 2024). Therefore, it is necessary for soft power research to further focus on individual-level perceptual experiences. How do netizens understand the global visibility of their country's traditional festivals? How do they evaluate the cultural attraction carried by these festivals? What statistical association does this evaluation have with their sense of group identity and digital participation?

It should be noted that “perceived global influence,” the focus of this study, must be explicitly defined as domestic netizens' subjective perception of overseas cultural visibility. It is not intended to serve as evidence of actual global influence, nor is it an objective measurement of international reception. Rather, it is a subjective judgment formed by domestic netizens based on their digital media experiences. Many respondents do not necessarily have direct intercultural contact experience; their understanding of issues such as “how foreigners view Chinese festivals” or “whether traditional festivals receive overseas attention” often comes from platform recommendations, news reports, short video content, comment section discussions, and peer sharing. Together, this information constitutes a digital observation window, allowing individuals to form impressions of the global dissemination status of traditional festivals even in the absence of direct overseas experience. Although this perception cannot replace evaluations by overseas audiences, it can represent the psychological projection of domestic netizens regarding their own culture's external image.

This issue is closely related to social identity and collective self-esteem. Social identity theory holds that an individual's evaluation of their belonging group is an important part of their self-understanding (Tajfel and Turner, 2004). When individuals believe their group possesses high value or a positive image in the external society, their evaluation of their group identity may also be higher. Existing research shows a close link between social media competence, digital citizenship, and collective self-esteem, and that collective self-esteem may play a mediating role between digital participation and identity (Islah and Helmi, 2024). Therefore, if individuals frequently encounter content about the overseas dissemination of traditional festivals in digital media and form a high evaluation of their global influence, this perception may be positively associated with their collective self-esteem.

Digital cultural empowerment provides another perspective. Social media provides ordinary users with the technical conditions to express, forward, and participate in cultural communication. Digital participation research indicates that self-efficacy, self-esteem, and social support are structurally associated with individuals' willingness and level of engagement with digital platforms (Kok Wah, 2025). Netizens posting photos of New Year customs, forwarding videos of overseas celebrations, or explaining the cultural meaning of festivals in comment sections—though merely daily interactions—may be accompanied by the psychological experience of “I can also participate in cultural communication.” The sense of digital cultural empowerment referred to in this study is precisely the psychological sense of efficacy individuals perceive in being able to participate in cultural expression, sharing, and communication through digital platforms. It does not imply that individuals will necessarily produce actual communicative behavior, but rather reflects their subjective evaluation of their digital participation ability and significance.

In this research framework, the level of digital contact and interaction becomes a variable worth noting. Liking, commenting, forwarding, and browsing content related to traditional festivals are not purely media usage behaviors; they may also relate to individuals' judgments of the festivals' global visibility, their evaluation of their own cultural group, and their feelings about the significance of online participation. Thus, the relationship between digital contact and interaction levels and perceived cultural influence, collective self-esteem, and the sense of digital cultural empowerment constitutes the core research concern of this paper.

At the same time, the media forms of digital communication are worth inclusion in the discussion. Currently, traditional festival content can be presented through text-and-image long articles, news reports, and knowledge cards, as well as through short videos, live broadcasts, vlogs, and cross-platform visual narratives. Audiovisual content usually possesses a stronger sense of presence and emotional contagion, while text-and-image content is more convenient for explaining cultural backgrounds and organizing festival knowledge. Short video communication research also indicates that dynamic audiovisual content may influence audience judgments through narrative transportation, emotional contagion, and persuasion effects (Liu and Deng, 2025; Zhu et al., 2023).

As intercultural experience affects how individuals understand community culture, local knowledge, and cultural differences (Louhapensang and Noobanjong, 2021), this study further examines whether overseas experience alters the association between digital interaction and perceived global influence. This leads to a specific question: Do netizens who prefer audiovisual modalities like short videos exhibit higher perceived global influence at the same interaction frequency? Or, in an environment where platform algorithms continuously integrate content forms, do differences in media modality no longer constitute a significant source of psychological difference? This question is both theoretically significant and closely related to practical communication.

Based on the above background, this study adopts a cross-sectional questionnaire survey method, using domestic netizens as a sample to examine the statistical associations among digital media interaction, media modality preference, perceived global influence, collective self-esteem, and the sense of digital cultural empowerment. Given the cross-sectional nature of the data, the mediation and serial models presented in this study represent statistical associations tested under a theoretical framework rather than strict causal structures.

The questions this paper hopes to answer include: Is the level of digital contact and interaction of domestic netizens around traditional festival content positively correlated with their perceived global influence? Does perceived global influence present a mediating association between digital interaction and collective self-esteem/digital cultural empowerment? Does media modality preference change the strength of the association between digital interaction and perceived global influence? Is there a serial association pattern consistent with the “cognition-emotion-empowerment” theoretical logic among digital interaction, perceived global influence, collective self-esteem, and the sense of digital cultural empowerment?

Analyzing these questions helps extend cultural soft power research from the level of macro communication effects to the micro-psychological level of online groups. More specifically, this study does not attempt to prove the objective degree of recognition traditional festivals have achieved globally, but focuses purely on how domestic netizens subjectively understand the possibility of being “seen” through digital media experiences and the structural correlation between this understanding and their cultural identity evaluation and sense of digital participation.

## 2 Theoretical basis and research hypotheses

### 2.1 Digital contact and interaction levels and perceived global influence

The digital media environment has changed the way individual's access information about traditional festivals. Festival content on social platforms is characterized by high frequency, fragmentation, and interactivity. Users can not only watch videos of Spring Festival celebrations abroad and read reports on Mid-Autumn cultural communication but also participate in information diffusion through likes, comments, and forwards. From the perspective of soft power research, cultural attraction is an important source of national influence (Nye, 1990); in the digital media environment, this attraction is increasingly presented through public interaction, symbolic exchange, and spontaneous communication on network platforms (Shahin and Huang, 2024; Yeo et al., 2024). Additionally, spontaneous interactions driven by cross-cultural social media influencers have been proven capable of actively assisting in the reconstruction of China's national image and cultural soft power (Liu Q., 2025).

Cultivation theory in communication emphasizes that there is an association between continuous media exposure and individuals' perception of social reality (Gerbner et al., 1986). In the social media environment, this contact is not just passive viewing but also includes active participation. Users who interact frequently with traditional festival content are generally more likely to repeatedly encounter similar information, such as overseas Chinese celebrating the Spring Festival, foreign tourists experiencing Dragon Boat customs, and discussions of Chinese festivals on international social platforms. This information may form a relatively stable impression in individuals' minds that traditional festivals possess a certain international visibility and cultural attraction.

From actual questionnaire scenarios, respondents are likely to mobilize their recent or long-term digital media experiences when answering questions like “whether traditional festivals have global influence.” If they frequently see related content on platforms and participate in liking, commenting, or forwarding, their evaluation of the global communication activity of festivals may be higher. What is discussed here is the correlation between digital contact and interaction levels and subjective perception, rather than the objective change in international influence by digital interaction.

Drawing upon these everyday digital engagement patterns, we formulate the first hypothesis:

H1: An individual's level of digital media contact and interaction with traditional festival-related content is significantly and positively correlated with their perception of the global influence of traditional festivals.

### 2.2 Mediating association of perceived global influence

Traditional festivals possess distinct group identity attributes. The reunion narrative of the Spring Festival, the home-country sentiment of the Mid-Autumn Festival, and the historical memory of the Dragon Boat Festival make them transcend general entertainment content and become important symbols of cultural belonging. When netizens perceive in digital space that these festivals receive overseas attention or are understood by intercultural groups, what they gain is not just a judgment at the information level but also something linked to their own evaluation as members of a cultural group.

The concept of collective self-esteem focuses on how individuals evaluate the social groups they belong to. Public collective self-esteem involves an individual's understanding of how the outside world views their group (Luhtanen and Crocker, 1992). In the digital environment, relevant studies also show that collective self-esteem can play a mediating role between digital citizenship, social media competence, and individual psychological evaluation (Islah and Helmi, 2024). The relationship between social media use and individual psychological outcomes is not a one-way linear one but is influenced by factors such as usage patterns, individual differences, and social contexts (Valkenburg et al., 2022). Intercultural social media research also shows complex associations among social media use, self-esteem, and cultural factors (Liu Z., 2025). In the context of this study, perceived global influence can be viewed as a subjective judgment regarding external evaluation. If respondents believe that Chinese traditional festivals possess attraction, recognition, and communication value globally, their collective self-esteem scores may also be higher. Such a relationship does not imply a one-way deterministic relationship between the two but indicates that they may present a close statistical link within the same psychological structure.

The sense of digital cultural empowerment is similarly related to this process. Network empowerment research shows that the internet provides individuals with space for expression, participation, and establishment of a sense of meaning, and is positively associated with a heightened subjective sense of efficacy in the digital environment (Amichai-Hamburger et al., 2008). Regarding traditional festival issues, if individuals believe that relevant cultural symbols already possess a certain international visibility, they may be more likely to understand their own online forwarding, explanation, and commenting as meaningful digital cultural participation. This sense of psychological efficacy may be positively associated with perceived global influence.

Therefore, this study places perceived global influence between digital interaction and collective self-esteem/digital cultural empowerment for investigation. Its theoretical implication is that netizens with higher levels of digital contact and interaction may exhibit higher perceived global influence, which in turn is statistically associated with their group identity evaluation and digital participation efficacy.

To test this psychological mechanism, we propose the following mediating pathways:

H2a: Perceived global influence presents a significant mediating association between “digital contact and interaction level” and “collective self-esteem.”

H2b: Perceived global influence presents a significant mediating association between “digital contact and interaction level” and “sense of digital cultural empowerment.”

### 2.3 Moderating association of media modality preference

Traditional festival content features multi-modal presentation on digital platforms. Text-and-image content is more suitable for explaining festival origins, ritual knowledge, and cultural symbols, while short videos and live broadcasts are easier for displaying festival scenes, ritual atmospheres, and character emotions. Although different modalities can all provide information on the global dissemination of festivals, their sense of presence and degree of emotional involvement may differ.

Media affordance theory holds that media forms influence users' information processing methods, interaction paths, and communication practices (Evans et al., 2017). Through visuals, music, editing, and scene organization, dynamic audiovisual content often more easily presents the festive atmosphere and cross-cultural participation scenes. Short video communication research shows that the narrative transportation, emotional contagion, and persuasion effects of audiovisual content are closely linked to users' attitude formation and judgment processes (Montag et al., 2021; Liu and Deng, 2025; Zhu et al., 2023). Therefore, among users who prefer audiovisual modalities such as short videos or live broadcasts, the positive association between digital contact and interaction levels and perceived global influence may be more pronounced. Especially in traditional festival communication, if scenes of overseas celebrations and foreign users experiencing Chinese festivals are presented in video form, they may leave a more direct impression on the audience.

However, the content environment of current social media platforms is already highly mixed. Much text-and-image content embeds videos, and short video comment sections carry a large amount of explanatory text; in actual use, users often encounter information across modalities rather than staying fixed in a single content form. Platform algorithms also continuously push similar themes based on interests, leading users of different modalities into similar information environments. Thus, whether media modality preference truly changes the relationship between digital interaction and perceived global influence cannot be judged solely by theoretical presets and still requires empirical data testing.

To empirically determine whether visual formats amplify this cognitive association, we posit:

H3: Media modality preference presents a moderating association in the positive relationship between “digital contact and interaction level and perceived global influence”; this positive association may be stronger among netizens who prefer audiovisual modalities.

### 2.4 Serial association from influence perception and collective self-esteem to digital cultural empowerment

Cultural psychological processes usually have certain levels. Individuals may first form a cognitive judgment of the external visibility of a cultural phenomenon, then generate an emotional evaluation at the group identity level based on this, and further manifest this as a judgment of their own participation ability and significance. Putting this idea into the context of digital communication of traditional festivals, perceived global influence can be understood as an evaluation at the cognitive level, collective self-esteem reflects affirmation at the identity and emotional levels, and digital cultural empowerment points to individuals' psychological efficacy judgment regarding online cultural participation. Psychological empowerment theory emphasizes that individuals' subjective judgments of their own ability, sense of meaning, and influence are important components of the empowerment experience (Spreitzer, 1995); network empowerment research further points out that the internet environment can provide individuals with space for expression, connection, and participation in public issues (Amichai-Hamburger et al., 2008). Existing digital participation research also points out that self-efficacy, self-esteem, and social support are important psychological resources affecting individuals' digital participation (Kok Wah, 2025). Therefore, if individuals perceive in digital space that their country's traditional festivals have high external visibility and thus form a more positive group identity evaluation, their sense of digital cultural empowerment may show a corresponding positive shift.

In actual platform use, this serial association is not difficult to understand. For instance, a netizen who frequently encounters content about the overseas dissemination of traditional festivals might be more inclined to agree that “traditional festivals possess global attraction.” Consequently, when they believe their country's festivals are being watched and discussed internationally, their positive evaluation as a cultural group member tends to be higher. When this group evaluation is positive, the individual's perceived meaningfulness in participating in cultural communication also exhibits a stronger associative pattern.

This study summarizes this structure as a “cognition-emotion-empowerment” serial association. Digital interaction is linked to perceived global influence, perceived global influence is linked to collective self-esteem, and collective self-esteem is linked to digital cultural empowerment. The sequential path proposed in this serial model is derived from the aforementioned theoretical logic, serving to test whether this conceptual structure aligns with the sample data.

Integrating these individual psychological evaluations into a cohesive structural framework, we test the final serial assumption:

H4: Perceived global influence and collective self-esteem present a serial mediating association between digital contact and interaction levels and the sense of digital cultural empowerment, i.e., there exists a statistical association path of DMU → INF → CSE → EMP.

**Figure 1:**
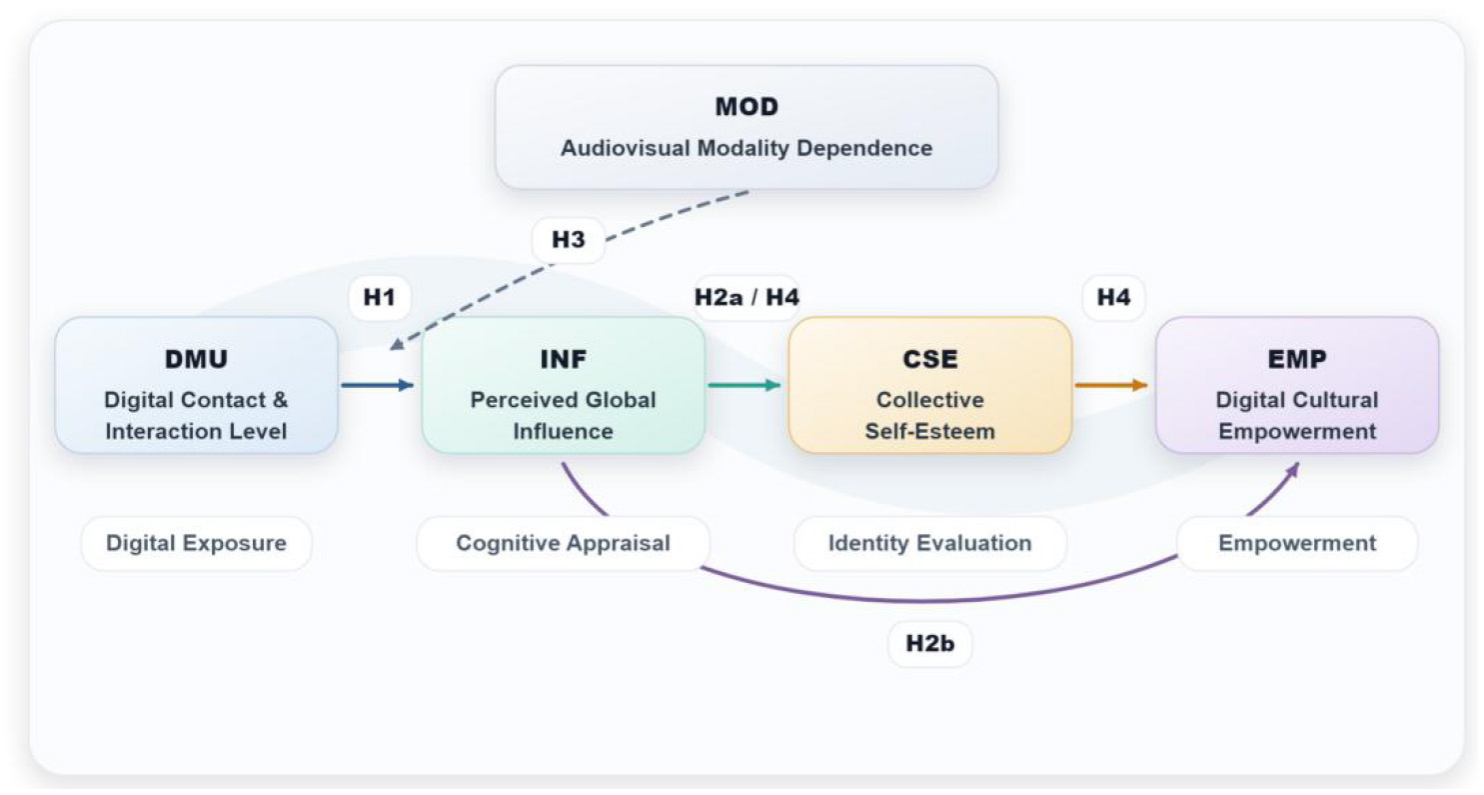
Theoretical model of the research. DMU, Digital contact and interaction level; INF, Perceived global influence; CSE, Collective self-esteem; EMP, Digital cultural empowerment; MOD, Audiovisual modality dependency. Solid arrows represent mediation and serial mediation hypothesis paths; dashed arrows represent moderation hypothesis paths. Paths in the figure represent statistical association structures under theoretical hypotheses and do not represent strict causal relationships.

Based on the above theoretical derivations, this study constructs a theoretical model among digital contact and interaction levels, perceived global influence, collective self-esteem, digital cultural empowerment, and audiovisual modality dependency. As shown in Figure 1, digital contact and interaction levels are placed at the start of the model, perceived global influence is viewed as a key cognitive variable connecting digital interaction experience and cultural-psychological evaluation, and collective self-esteem further connects influence perception and digital cultural empowerment. Meanwhile, this study takes audiovisual modality dependency as a moderating variable to test whether it changes the association strength between digital contact and interaction levels and perceived global influence. It should be noted that because this study adopts a cross-sectional design, the paths in the figure represent a statistical association structure based on theoretical hypotheses rather than causal sequences in a strict sense.

## 3 Research method

### 3.1 Participants and procedure

This study used a questionnaire survey method for cross-sectional data collection. The data collection period was from October to December 2025. This study employed a convenience sampling method, releasing the questionnaire link online via the “Wenjuanxing” platform and disseminating it through social media channels to recruit domestic netizens aged 18 and older. To reduce the risk of repeated answers, the questionnaire platform enabled a repeat submission restriction function. Respondents were required to read an informed consent form and confirm they were at least 18 years old and participated voluntarily before officially filling out the questionnaire. The first page of the questionnaire explicitly stated that the research was for academic purposes only, all responses were collected anonymously and de-identified, and participants could withdraw at any time. The complete questionnaire content is in Supplementary Data Sheet 1. A total of 1,600 initial questionnaires were collected. In the data preprocessing stage, invalid samples with abnormally short response times (below a strict 90-s minimum cognitive threshold) or those that failed the internal Attention Check were deleted according to preset screening criteria. The attention check question was placed in the perceived global influence question group, requiring respondents to select “Strongly Disagree” as prompted; those who did not comply were eliminated. The attention-check failure and rapid-response exclusions cumulatively accounted for a 3.75% failure rate (60 respondents). Missing data were inherently prevented by configuring the survey logical rules to strictly require responses for all mandatory substantive items prior to submission. Finally, 1,540 valid samples were retained. The detailed demographic characteristics of these valid samples are summarized in Table 1. The names, assignment methods, and coding rules of each variable in the final dataset are detailed in Supplementary Data Sheet 2.

**Table 1:** Demographic characteristics of the sample (*N* = 1,540).

Variables	Category	Frequency (*n*)	Percentage (%)
Gender	Male	730	47.40
	Female	810	52.60
Age (years)	18–25	561	36.43
	26–35	599	38.90
	36–45	231	15.00
	46 and above	149	9.67
Education level	High school or below	171	11.10
	Junior college	211	13.70
	Bachelor's degree	925	60.07
	Master's degree or above	233	15.13
Overseas experience	Yes (≥ 3 months)	238	15.45
	No	1,302	84.55

### 3.2 Measures

The psychological and perceptual assessment variables included in this study primarily adopted or were adapted from established domestic and international scales, with appropriate phrasing adjustments combined with the context of digital communication of traditional Chinese festivals. To support transparency and scientific replication, the exact wording of all 12 primary scale items is fully provided in Supplementary Data Sheet 1. Except for the average daily digital media usage time item, which used 4-level categorical scoring, the core psychological and perceptual items primarily used 5-point Likert scoring, where 1 means “Strongly Disagree” and 5 means “Strongly Agree.”

Digital Media Usage and Engagement (DMU): Digital media usage characteristics were measured by two items: first, the average daily social media usage time of respondents, with a value range of 1 to 4, corresponding to “within 1 hour,” “1–3 hours,” “3–5 hours,” and “more than 5 hours”; second, the frequency with which respondents actively read, liked, commented on, or forwarded content related to “Chinese traditional festivals” on social media in the past year, with a value range of 1 to 5, corresponding to “Never” to “Always.” This variable reflects respondents' level of digital media contact and interaction around traditional festival content. These two items were aggregated into a composite DMU construct to capture a holistic picture of individuals' digital immersion. The justification for this aggregation rests on the logic that general social media use establishes the foundational base-rate probability of encountering cultural content, while festival-specific interaction captures active behavioral engagement; combining them thus provides a more robust proxy for overall digital habituation.

Digital Modality Tendency (MOD): Digital modality tendency was used to measure whether respondents, compared to traditional text-and-image content, were more inclined to rely on dynamic images combining audio and visuals, such as short videos, live broadcasts, and vlogs, to obtain information related to the global dissemination of traditional festivals. This dimension included two items, examining respondents' degree of dependency on short videos/live broadcasts for obtaining information and their evaluation of the intuitiveness of audiovisual content in conveying the overseas influence of traditional Chinese culture.

Perceived Global Influence (INF): Perceived global influence was developed based on soft power theory and national image communication models to determine respondents' overall evaluation of the attraction of traditional Chinese festival cultural symbols, value empathy, and digital media display efficacy (Buhmann and Ingenhoff, 2015; Nye, 1990). This dimension contained 4 formal scoring items, involving discussions or celebrations of Chinese traditional festivals by foreign netizens, the attraction of traditional festival cultural symbols to overseas audiences, overseas audiences' understanding and empathy for festival values, and the effectiveness of digital media in displaying the cultural soft power of traditional festivals. Additionally, 1 attention check question was set in this group for data quality control and was not included in formal statistical analysis.

Collective Self-Esteem (CSE): Collective self-esteem measurement was taken from the “Public Collective Self-Esteem” dimension of Luhtanen and Crocker's (1992) Collective Self-Esteem Scale, with phrasing adjustments combined with the digital communication context of traditional festivals. This variable was used to measure netizens' sense of group self-affirmation based on their perception of external evaluations of their cultural group, specifically including their perception of how the international internet society evaluates Chinese culture and the cultural pride they feel when seeing traditional festivals “go global.”

Digital Cultural Empowerment (EMP): Digital cultural empowerment draws on the concepts of psychological empowerment and network empowerment to measure netizens' psychological sense of efficacy in perceiving whether they can contribute to the dissemination of traditional Chinese culture through digital contact and interaction (Amichai-Hamburger et al., 2008; Spreitzer, 1995). This variable included two items, measuring whether respondents believe ordinary netizens can contribute to the global dissemination of traditional Chinese culture through digital contact and interaction, and whether high-quality traditional festival communication content would stimulate their willingness to actively disseminate culture. Thus, this variable focuses on reflecting respondents' cognitive sense of empowerment regarding their digital cultural participation ability and their comprehensive tendency toward externalized communication willingness.

### 3.3 Data analysis strategy

Research data were statistically processed using SPSS 27.0 and the PROCESS v4.0 macro. After the raw data were assigned values, named, and cleaned, invalid samples with abnormal response times or failed attention checks were eliminated. Subsequently, descriptive statistics and normality tests were performed on each observed variable, reporting indicators such as mean, standard deviation, skewness, and kurtosis.

The measurement model was tested through exploratory factor analysis and confirmatory factor analysis. Reliability was primarily judged based on the Cronbach's α coefficient and composite reliability (CR); convergent validity was judged based on standardized factor loadings and average variance extracted (AVE); discriminant validity was evaluated according to the Fornell-Larcker criterion. Simultaneously, this study used the Harman single-factor test and single-factor confirmatory factor analysis to screen for common method bias. Regarding variable relationship testing, Pearson correlation analysis was used to examine the correlations among digital media interaction, media modality preference, perceived global influence, collective self-esteem, and digital cultural empowerment, and independent sample *t*-tests and one-way analysis of variance (ANOVA) were used to compare differences across different demographic groups on core variables.

Hypothesis testing was primarily completed using the PROCESS v4.0 macro, with 5,000 bootstrapping samples to obtain confidence intervals for judging effect significance (Hayes, 2022). Specifically, Model 4 was used to test mediation effects, Model 6 for serial mediation paths, and Model 7 for the moderated mediation model. Demographic variables such as gender, age, and overseas experience were controlled in the model analysis. Furthermore, this study used multi-group analysis (MGA) to further examine the stability of core paths between groups with and without overseas experience.

## 4 Results

### 4.1 Data preprocessing and common method bias test

After obtaining 1,540 valid questionnaires, this study first screened and processed extreme outliers using the Z-score method to ensure overall data quality. Subsequently, structured normality tests were conducted on each observed indicator. As shown in Table 2, the means of the measurement items ranged from 3.125 to 3.790, and standard deviations ranged from 0.812 to 0.937, showing good dispersion.

**Table 2:** Descriptive statistics and normality tests of observed variables (*N* = 1,540).

Construct	Item	Mean	*SD*	Skewness	Kurtosis
Digital contact and interaction (DMU)	Q6_DMU1	3.125	0.812	−0.546	−0.481
	Q7_DMU2	3.192	0.937	−0.019	−0.306
Media modality (MOD)	Q8_1_MOD1	3.583	0.907	−0.211	−0.393
	Q8_2_MOD2	3.578	0.894	−0.178	−0.418
Perceived global influence (INF)	Q9_1_INF1	3.686	0.900	−0.232	−0.513
	Q9_2_INF2	3.696	0.890	−0.284	−0.382
	Q9_4_INF3	3.668	0.895	−0.238	−0.437
	Q9_5_INF4	3.675	0.885	−0.201	−0.499
Collective self-esteem (CSE)	Q10_1_CSE1	3.778	0.895	−0.400	−0.252
	Q10_2_CSE2	3.790	0.889	−0.404	−0.247
Digital cultural empowerment (EMP)	Q10_3_EMP1	3.488	0.932	−0.258	−0.302
	Q10_4_EMP2	3.477	0.920	−0.116	−0.429

Further statistical findings showed that the absolute values of the skewness coefficients of each observed variable ranged from 0.019 to 0.546, and the absolute values of the kurtosis coefficients ranged from 0.247 to 0.513. Since the absolute values of skewness and kurtosis for all indicators were well below the statistical threshold of 2.0, and most were less than 1.0, it indicated that the items did not show serious deviation from normal distribution, basically meeting the distribution requirements for using the Maximum Likelihood (ML) method for measurement model estimation.

Based on the potential risks of self-report data, in the common method bias test, the Harman's unrotated single-factor test results showed that the variance explanation rate of the first factor was 37.34%, lower than the empirical threshold of 40%.

To further evaluate the potential impact of common method bias, this study additionally used Single-factor CFA for testing. In this procedure, all 12 indicators of the core constructs were forced to load on a single latent factor, and the model fit was calculated. As shown in Table 3, the single-factor model performed poorly (χ^2^/*df* = 36.71, CFI = 0.701, TLI = 0.635, RMSEA = 0.152), far below the fit level of the five-factor baseline model. In contrast, the five-factor original model exhibited excellent fit (χ^2^/*df* = 1.016, CFI = 1.000, TLI = 1.000, RMSEA = 0.003). This indicates that the probability of serious common method bias caused by a single factor is extremely low.

**Table 3:** Single-factor CFA fit comparison test for common method bias (*N* = 1,540).

Models	χ^2^	*df*	χ^2^/*df*	CFI	TLI	RMSEA
Single-factor CFA	1,982.21	54	36.71	0.701	0.635	0.152
Original five-factor model	44.69	44	1.016	1.000	1.000	0.003

The single-factor model loads all core measurement items on the same latent factor; the original five-factor model includes five latent variables: digital contact and interaction level, media modality, perceived global influence, collective self-esteem, and digital cultural empowerment.

### 4.2 Reliability and validity of the measurement model

**Figure 2:**
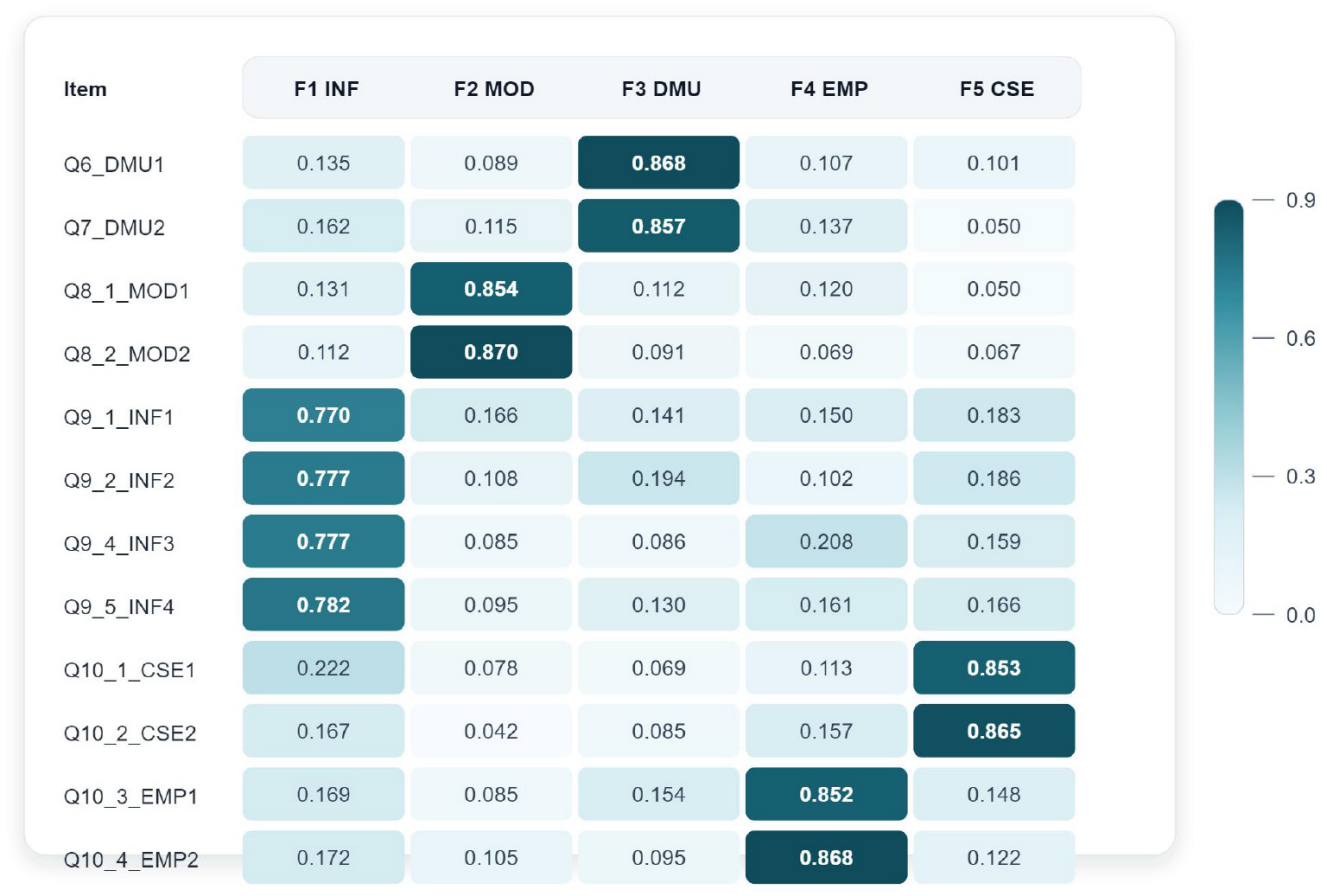
Rotated loading heatmap of exploratory factor analysis. Extraction method was principal component analysis, rotation method was Varimax orthogonal rotation; color depth indicates the size of factor loadings, dark cells and bold values indicate the primary loadings of each item on its theoretical factor. KMO = 0.831, Bartlett's test of sphericity χ^2^ = 6,490.22, *p* < 0.001; 5 factors cumulatively explained 76.33% of the variance.

To further test the suitability of the measurement structure, this study first conducted an exploratory factor analysis (EFA) on the 12 core measurement items. The results showed a KMO value of 0.831, and the Bartlett's test of sphericity was significant (χ^2^ = 6490.22, *p* < 0.001), indicating the data was suitable for factor analysis. Using the principal component extraction method and Varimax orthogonal rotation, 5 factors were extracted according to the theoretical structure, with a cumulative variance explanation rate of 76.33%. The rotated factor loadings showed that each item had a high loading on its theoretical factor, with primary loadings ranging from 0.770 to 0.870, and no obvious cross-factor high loading phenomena occurred. This result is basically consistent with the five-factor structure preset in this study; specific results are shown in Figure 2.

Based on the exploratory factor analysis, this study further used Maximum Likelihood estimation for confirmatory factor analysis (CFA) to test the structural validity of the five-factor measurement model. The results showed that the five-factor measurement model exhibited excellent model fit (χ^2^/*df* = 1.016, RMSEA = 0.003, CFI = 1.000, TLI = 1.000).

In terms of reliability, the Cronbach's α coefficients of each latent variable were between 0.71 and 0.85, and the composite reliability (CR) was greater than 0.70, indicating good internal consistency. As shown in Table 4, the standardized factor loadings for digital contact and interaction (DMU), media modality (MOD), perceived global influence (INF), collective self-esteem (CSE), and digital cultural empowerment (EMP) all reached acceptable levels, and the average variance extracted (AVE) for each latent variable exceeded 0.50, indicating good convergent validity.

**Table 4:** Confirmatory factor analysis, composite reliability, and convergent validity.

Constructs	Items	Standardized loadings (λ)	*p-value*	Cronbach's α	CR	AVE
DMU	Q6_DMU1	0.749	—	0.742	0.747	0.597
	Q7_DMU2	0.795	^***^			
MOD	Q8_1_MOD1	0.788	—	0.719	0.721	0.564
	Q8_2_MOD2	0.712	^***^			
INF	Q9_1_INF1	0.778	—	0.849	0.849	0.585
	Q9_2_INF2	0.770	^***^			
	Q9_4_INF3	0.750	^***^			
	Q9_5_INF4	0.761	^***^			
CSE	Q10_1_CSE1	0.796	—	0.754	0.755	0.606
	Q10_2_CSE2	0.761	^***^			
EMP	Q10_3_EMP1	0.812	—	0.770	0.770	0.627
	Q10_4_EMP2	0.771	^***^			

*** p < 0.001. The first item of each latent variable was fixed as a marker variable, so its significance test is not reported separately. Overall model fit indices: χ^2^ = 44.69, df = 44, χ^2^/df = 1.016, RMSEA = 0.003, CFI = 1.000, TLI = 1.000.

Regarding discriminant validity, this study judged based on the Fornell-Larcker criterion. This criterion holds that if the square root of the AVE for each latent variable is higher than its correlation coefficient with other latent variables, the model can be considered to have good discriminant validity (Fornell and Larcker, 1981). As shown in Table 5, the square root of the AVE for each latent variable was greater than its correlation coefficient with other latent variables, supporting the discriminant validity of the measurement model in this study.

**Table 5:** Discriminant validity test (Fornell-Larcker criterion).

Variables	1	2	3	4	5
1. DMU	**0.773**				
2. MOD	0.266^***^	**0.751**			
3. INF	0.375^***^	0.315^***^	**0.765**		
4. CSE	0.228^***^	0.184^***^	0.454^***^	**0.778**	
5. EMP	0.319^***^	0.256^***^	0.424^***^	0.346^***^	**0.792**

Bold diagonal data are the square root of the AVE for each latent variable; off-diagonal data are Pearson correlation coefficients; discriminant validity is supported when the square root of the AVE is higher than corresponding correlations.

*** p < 0.001.

### 4.3 Descriptive statistics, correlations, and group difference characteristics

Overall descriptive statistics showed that domestic netizens' evaluation of collective self-esteem was high (*M* = 3.78), and they also held positive perceptions of the global influence of our traditional festivals (*M* = 3.68). Pearson correlation analysis mapped out the statistical associations supporting the baseline hypotheses as shown in Table 6: digital contact and interaction levels showed significant positive association sequences with perceived global influence (*r* = 0.375, *p* < 0.001), collective self-esteem (*r* = 0.228, *p* < 0.001), and digital cultural empowerment (*r* = 0.319, *p* < 0.001).

**Table 6:** Summary of means, standard deviations, and correlations of latent variables.

Variables	Mean	SD	1	2	3	4	5
1. DMU	3.16	0.78	1				
2. MOD	3.58	0.80	0.266^***^	1			
3. INF	3.68	0.74	0.375^***^	0.315^***^	1		
4. CSE	3.78	0.80	0.228^***^	0.184^***^	0.454^***^	1	
5. EMP	3.48	0.84	0.319^***^	0.256^***^	0.424^***^	0.346^***^	1

*** p < 0.001; diagonal is 1. Means are the average score after dimension aggregation.

To further identify distribution differences of core variables across demographic groups, this study compared intergroup differences in gender, overseas experience, and education level on perceived global influence (INF) and collective self-esteem (CSE). As shown in Table 7, differences in gender and education level on INF and CSE did not reach significance; differences in overseas experience on INF were also non-significant (*t* = −1.28, *p* = 0.202), but showed a trend toward significance on CSE (*t* = −1.95, *p* = 0.052). Overall, mean differences of core variables across demographic groups were limited; relevant demographic variables will be included as control variables in subsequent models.

**Table 7:** Comparison of sample differences in core perceptions by demographic characteristics (*N* = 1,540).

Variable attribute	Category	*N*	INF Mean (*SD*)	Difference test	CSE Mean (*SD*)	Difference test
Gender	Male	730	3.71 (0.73)	*t* = 1.56	3.79 (0.80)	*t* = 0.50
	Female	810	3.65 (0.75)	*p* = 0.119	3.77 (0.80)	*p* = 0.616
Overseas	Yes	238	3.62 (0.81)	*t* = −1.28	3.69 (0.80)	*t* = −1.95
	No	1,302	3.69 (0.73)	*p* = 0.202	3.80 (0.80)	*p* = 0.052
Education	High school or below	171	3.69 (0.71)	*F* = 1.21	3.77 (0.80)	*F* = 1.16
	Junior college	211	3.68 (0.71)	*p* = 0.304	3.76 (0.79)	*p* = 0.326
	Bachelor's	925	3.66 (0.76)		3.77 (0.81)	
	Master's or above	233	3.76 (0.69)		3.87 (0.78)	

Gender and overseas experience used independent sample t-tests; education level used one-way ANOVA. Overseas experience approached the conventional significance threshold for CSE (p = 0.052) but remained strictly non-significant. Subsequent path models include gender, age, and overseas experience as control variables.

### 4.4 Hypothesis mechanism testing: mediation and moderation analysis based on hierarchical regression and PROCESS

This study first used hierarchical multiple regression analysis to determine the main effects of research hypotheses, then leveraged PROCESS v4.0 (Model 4 and Model 7) to examine the requirements for establishing mediation and moderated framework mechanisms:

#### 4.4.1 Main effect and mediation effect (H1 & H2a)

To test H1, this study first examined the association of digital contact and interaction level (DMU) on perceived global influence (INF). Results showed that DMU significantly and positively associated with INF, with a non-standardized regression coefficient of *B* = 0.354, *p* < 0.001, indicating that the higher the respondents' level of digital contact and interaction around traditional festival content, the stronger their perception of the global influence of traditional festivals; H1 was supported.

Subsequently, this study tested the total effect of digital contact and interaction levels with collective self-esteem (CSE) as the dependent variable. As shown in Table 8, in hierarchical regression, after placing control variables (gender, age, overseas experience) into Block 1, and the DMU variable into Block 2, the overall model explanatory power significantly improved (Δ*R*^2^ = 0.050, *p* < 0.001). At this point, DMU had a significant positive association with CSE (non-standardized coefficient *B* = 0.229, *p* < 0.001), indicating a significant positive association between digital contact and interaction level and collective self-esteem, providing a statistical basis for further testing mediation effects.

**Table 8:** Hierarchical regression analysis of digital contact and interaction level on collective self-esteem.

Predictors	Block 1 (Control model)		Block 2 (Main effect model)	
	*B*	*t*-value	*B*	*t*-value
Control variables				
Gender	0.027	0.65	−0.009	−0.23
Age	0.029	1.45	0.019	0.99
Overseas	−0.026	−0.46	0.071	1.29
Independent variable				
DMU			0.229^***^	8.96
Model summary				
*R* ^2^	0.004		0.054	
Δ*R*^2^			0.050^***^	

Coefficient B is the non-standardized regression coefficient;

*** p < 0.001.

**Figure 3:**
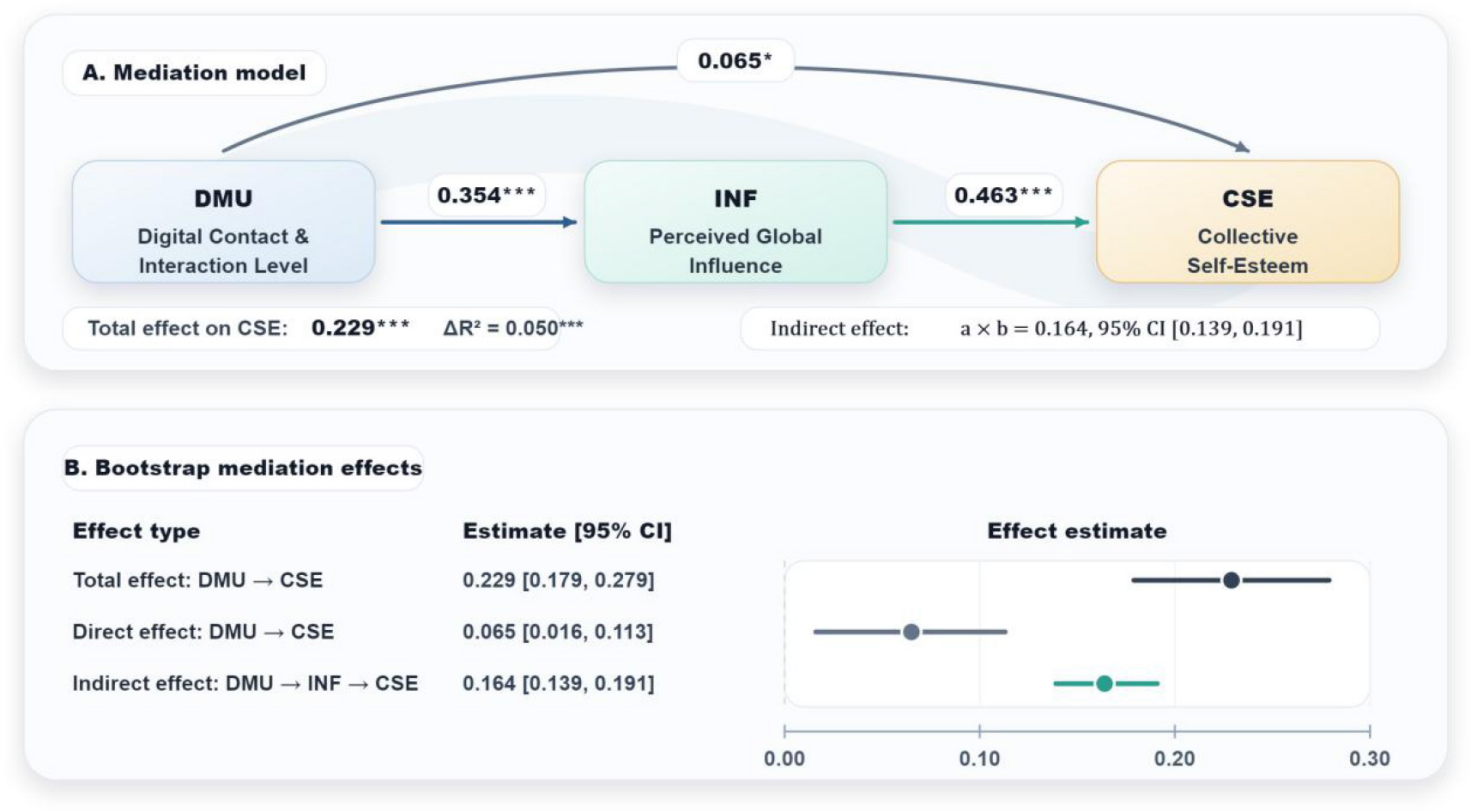
Results of the mediation effect of perceived global influence. **(A)** Shows mediation path coefficients among digital contact and interaction level (DMU), perceived global influence (INF), and collective self-esteem (CSE); **(B)** shows total effect, direct effect, and indirect effect along with 95% confidence intervals. Path coefficients and effect values are non-standardized. Indirect effect is estimated based on 5,000 Bootstrap samples; confidence intervals not containing 0 indicate effect significance. *p < 0.05, ***p < 0.001.

Further using PROCESS Model 4 to test the mediating role of perceived global influence (INF) between digital contact and interaction level (DMU) and collective self-esteem (CSE), results are shown in Figure 3. Bootstrapping 5,000 samples showed that DMU significantly and positively associated with INF (*a* = 0.354, *p* < 0.001), and INF also significantly and positively associated with CSE (*b* = 0.463, *p* < 0.001). After controlling for INF, the direct effect of DMU on CSE remained significant [direct effect = 0.065, 95% CI (0.016, 0.113)]. Simultaneously, the indirect effect of INF was 0.164, with a 95% confidence interval of [0.139, 0.191], not containing 0. Thus, perceived global influence presents a significant partial mediating association between digital media contact and interaction level and collective self-esteem; H2a was supported.

#### 4.4.2 Moderation mechanism test (H3)

This study used PROCESS v4.0 (Model 7) to conduct an empirical test of the moderating role of media modality preference (MOD). Statistical results showed that the coefficient of the interaction term between digital contact and interaction level (DMU) and media modality (MOD) on the mediating variable (INF) did not reach the significance level (*B* = 0.023, *t* = 0.853, *p* = 0.394 > 0.05).

Further examining the Index of Moderated Mediation, the result was 0.011, with a corresponding 95% Bootstrap confidence interval of [−0.012, 0.035]. Since the interval contains 0, it indicates that this study did not observe a significant moderated mediation effect. This suggests that the degree of audiovisual modality dependency did not significantly change the strength of the association between digital contact and interaction level and perceived global influence; conditional indirect effects under different modality dependency levels showed no significant differences, as shown in Table 9. Therefore, the data did not support hypothesis H3.

**Table 9:** Conditional effect analysis of moderated mediation model (PROCESS Model 7).

Audiovisual modality dependency (MOD)	Conditional indirect effect	Boot *SE*	Boot LLCI	Boot ULCI
Low preference (−1 *SD*)	0.129	0.015	0.099	0.158
Mean level (Mean)	0.138	0.013	0.111	0.165
High preference (+1 *SD*)	0.146	0.017	0.115	0.181
Index of moderated mediation				
Index	0.011	0.012	−0.012	0.035

The confidence interval of the index [−0.012, 0.035] contains 0, indicating no significant strengthening or weakening difference in the first half of the mediation path.

### 4.5 Serial mediation test based on PROCESS Model 6 (H4 & H2b)

To further test the serial association structure in the theoretical model, this study placed collective self-esteem (CSE) between perceived global influence and digital cultural empowerment (EMP), constructing a serial mediation model. Using PROCESS v4.0 (Model 6) for 5,000 bootstrapping tests, statistical results in Table 10 show that the total indirect effect was significant [effect value of 0.153, 95% CI (0.131, 0.191)], and the confidence intervals of the three specific indirect paths did not contain 0. Specific path performances were as follows:

**Table 10:** Serial mediated effects analysis of digital contact and interaction on empowerment sense.

Indirect pathways	Effect	Boot SE	Boot LLCI	Boot ULCI
Total indirect	0.153	0.015	0.131	0.191
Ind1: DMU → INF → EMP	0.110	0.012	0.085	0.131
Ind2: DMU → CSE → EMP	0.012	0.006	0.003	0.023
Ind3: DMU → INF → CSE → EMP	0.031	0.005	0.021	0.041

Based on 1,540 samples, 5,000 bootstraps; effect values are non-standardized coefficients.

Path 1 (DMU → INF → EMP): Effect value was 0.110, 95% CI [0.085, 0.131]; confidence interval does not contain 0, indicating perceived global influence plays a significant mediating role between digital contact and interaction level and digital cultural empowerment; H2b was supported. Path 2 (DMU → CSE → EMP): Effect value was 0.012, indicating collective self-esteem also exists as a parallel mediator between interaction behavior and empowerment. Path 3 Serial Path (DMU → INF → CSE → EMP): Effect value was 0.031, with a confidence interval of [0.021, 0.041], statistically supporting the serial mediation association proposed in H4.

The statistical significance of this serial path supports the “cognitive perception—psychological identity—efficacy externalization” association path proposed in this study. However, given this study's cross-sectional data, this result should be understood as a serial mediation in the sense of statistical association. While the data fit the proposed theoretical sequence, it does not confirm a strict causal or chronological mechanism.

### 4.6 Multi-group comparison of core path: heterogeneity analysis based on overseas experience

To further examine the stability of the core path “Digital Contact and Interaction Level → Influence Perception” across group characteristics, this study selected “Overseas Experience” as a grouping variable, using multi-group comparison analysis to contrast differences in coefficients between groups with continuous overseas experience of 3 months or more (*n* = 238) and groups without overseas experience (*n* = 1,302).

Statistical results in Table 11 show: in the group without overseas experience, the non-standardized regression coefficient of digital contact and interaction level on influence perception was 0.357 (*p* < 0.001); in the group with overseas experience, the coefficient was 0.343 (*p* < 0.001). Through significance testing of the difference between the two regression coefficients (*Z*-test), the result showed *Z* = 0.208, *p* = 0.835 > 0.05, failing to pass the significance threshold.

**Table 11:** Multi-group comparison results of the core path (DMU → INF) (*N* = 1,540).

Group	*n*	Coefficient (*B*)	*SE*	*t*-value	*p*	*Z*-score	Significant Difference (*p*)
No overseas experience	1,302	0.357	0.024	14.875	^***^	0.208	0.835
With overseas experience	238	0.343	0.063	5.444	^***^		

Target path for testing is DMU → INF;

*** p < 0.001.

This result indicates that the positive association between digital contact and interaction level and perceived global influence did not differ significantly due to whether individuals had overseas experience.

## 5 Discussion

### 5.1 Summary of main findings

Through a cross-sectional survey, this study explored the perceptual characteristics of domestic netizens regarding the global influence of Chinese traditional festivals and their association with individual internet psychology. Descriptive analysis showed that netizens gave high evaluations to both collective self-esteem and the sense of digital cultural empowerment. Demographic difference tests further showed that intergroup differences in gender, education level, and overseas experience on perceived global influence and collective self-esteem were generally limited; among them, overseas experience showed a trend toward significance in the collective self-esteem dimension. This suggests that different intercultural experience groups may have certain differences in cultural identity evaluation, but this result still requires further verification in subsequent studies.

Core model testing presented high statistical correlations with baseline hypotheses. Data showed that respondents' digital media contact and interaction levels were significantly positively correlated with perceived global influence. After controlling for demographic variables, influence perception exhibited a significant incomplete mediating role between network interaction and collective self-esteem/digital cultural empowerment. This finding echoes the recent research trend of soft power measurement shifting from macro national image to spontaneous public action and network interaction (Shahin and Huang, 2024; Yeo et al., 2024).

However, current data did not provide statistical support for the moderating role of audiovisual modality dependency (H3) (*p* = 0.394). Analysis showed that regardless of whether netizen groups' media dependency leaned toward dynamic audiovisuals (such as short videos) or traditional static text-and-images, the association slope between digital media contact and interaction level and perceived global influence did not show a significant difference. The non-significance of this modality moderation may imply that in today's highly homogenized network ecology driven by algorithm recommendation, the core element associated with people's cultural perception increment lies in the involvement of interaction itself (i.e., frequency and duration) rather than a single content presentation form. Importantly, the lack of significant moderation by audiovisual modality or overseas experience does not necessarily mean that media format or intercultural experience is unimportant in shaping cultural psychology. Instead, this non-significance may primarily reflect the inherent limitations in how these constructs were measured (e.g., relying on self-reported, aggregated modality preferences). Future research utilizing granular experimental designs or objective platform-algorithm tracking might still uncover more nuanced modality effects.

Further, through a serial mediation model, this study presented an associative path from cognitive perception and identity evaluation to empowerment experience. Data showed that there is not only a direct association between netizens' digital interaction and empowerment sense but also a serial association pattern via perceived global influence and collective self-esteem. This result provides empirical support for understanding the psychological associative structure of digital global influence perception. Furthermore, it is important to acknowledge that in cross-sectional mediation analysis, mathematically equivalent models (e.g., reversing the path to EMP → CSE → INF) could also yield significant statistical fits. Therefore, the directionality of our serial model is primarily grounded in the sequential logic of classical psychological empowerment theory (cognition → emotion → efficacy) rather than an observed temporal order.

Multi-group analysis further showed that the positive association between digital contact and interaction level and perceived global influence did not differ significantly between groups with and without overseas experience. This result may reflect that in a highly digitized media environment, individuals' subjective perception of the global dissemination of traditional festivals does not rely entirely on actual overseas experience but is closely related to their daily digital media contact and interaction experience.

### 5.2 Theoretical implications

The theoretical discussion in this paper mainly focuses on the following two points. First, this study attempts to place “cultural soft power,” usually used as a macro-political indicator, within the micro-empirical associative dimension of cyberpsychology. National image communication research emphasizes that cultural image is an important part of national image and is closely related to public evaluations of national attraction and value identity (Buhmann and Ingenhoff, 2015). This study further suggests that from the perspective of an interconnected world, perceived global influence can also be viewed as a social dynamic phenomenon manifested in domestic online groups. It has a significant positive statistical link with individuals' collective self-esteem construction, enriching the cross-sectional application of this theory in the digital dimension.

Second, through the serial mediation model, this study tested the “cognition-emotion-empowerment” theoretical path model among global cultural influence perception, collective self-esteem, and empowerment sense using cross-sectional data. This provides an empirical model for understanding the associative structure among cultural influence perception, group identity evaluation, and digital cultural empowerment, expanding the digital research boundaries of cultural psychology.

### 5.3 Practical implications

The data distribution characteristics of this study have certain reference significance for traditional culture communication paths in the digital era. Based on the associative results of regression analysis, given that media presentation modality did not exhibit a significant leverage difference, cultural communication practitioners may not need to obsess over converting all texts into high-cost short video modalities. Although relevant short video research shows that audiovisual content has communication potential in narrative transportation, emotional contagion, and persuasion effects (Liu and Deng, 2025; Zhu et al., 2023), results of this study show that interaction frequency itself may better explain statistical variations in influence perception than media form. Conversely, relevant strategies should focus on establishing effective “user interaction incentive mechanisms.” Since interaction frequency itself is closely related to group identity, exploring how to mobilize the public's substantive participation in information interaction for cultural “going global” through comments, hashtags, etc., should be viewed as a current priority path. Based on the serial mediation conclusion, communication agencies should realize that enhancing people's sense of cultural empowerment cannot be separated from the cultivation of their “collective self-esteem.” Communication content should not only present specific situations of traditional festivals being watched, discussed, and understood in international fields but also stimulate audiences' sense of group honor through emotional resonance. When netizens experience the psychological progression from “seeing influence” to “accompanying pride,” this psychological connection tends to be more closely associated with active cultural communication willingness in digital space.

## 6 Limitations and future directions

Considering the existing cross-sectional research design, the interpretation of data in this study should remain objective and restrained.

(1) Inherent limitations of cross-sectional data and alternative models: Although this study derived mediation and serial mediation models based on literature, data were extracted from a single point in time. The model reveals only cross-sectional associations between variables and cannot establish strict temporal sequences or causal mechanisms. Specifically, alternative theoretical models are highly plausible; for instance, individuals with inherently high collective self-esteem might proactively seek out more digital cultural engagement or project higher perceived cultural visibility. Future research should introduce cross-lagged panel models or longitudinal tracking designs to further clarify the objective occurrence sequence of these variable associations.
(2) Potential risk of common method bias: Although this study reported Harman and single-factor CFA *post-hoc* indices within safe limits, these retrospective statistical techniques cannot entirely rule out method artifacts. Because the entire model indicators rely on cross-sectional self-report scales, the limitation of puffed associations induced by common method variance is difficult to completely filter out. Future validations require more robust procedural controls, such as temporal separation of measurements or integrating objective platform behavioral logs.
(3) Limitations of single cultural context samples: The sample is limited to the context of domestic netizens in China, reflecting the “in-group's projected perception of its own culture being accepted externally.” This perception may not completely overlap with evaluations by real overseas audiences. Future research could consider distributing scales to audiences with different cultural backgrounds for parallel measurement comparison.
(4) Confounding factors of the macro environment: Netizens' “subjective perception of global influence” belongs to a variable extremely sensitive to the external society; such uncontrollable factors (like sudden international cultural exchange events) may unavoidably form short-term fluctuation interference during data collection.
(5) Limitations of convenience sampling and generalizability: Given the reliance on a digitally-recruited convenience sample, the empirical results primarily map the psychological structural associations of the surveyed participants. Consequently, specific index scores and associative findings should be interpreted with caution and cannot be strictly generalized to represent the entire, highly diverse population of all Chinese netizens.

## 7 Conclusion

Based on 1,540 cross-sectional survey data, this study found: individuals' level of digital contact and interaction is significantly and positively correlated with their perception of the global influence of Chinese traditional festivals; in terms of associative mechanisms, perceived global influence and collective self-esteem together constitute an incomplete serial mediating relationship pointing toward the sense of digital cultural empowerment (DMU → INF → CSE → EMP). Simultaneously, neither audiovisual modality dependency nor overseas experience produced significant moderating effects on this core associative path. Overall, in the digital interconnection environment, the subjective perception of cultural soft power of traditional festivals is increasingly intertwined with netizens' digital contact and interaction experiences, group identity evaluations, and sense of digital cultural participation efficacy. This study provides cross-sectional empirical evidence for understanding the cultural-psychological mechanisms in the digital communication of traditional festivals.

## Data Availability

The original contributions presented in the study are included in the article/Supplementary material, further inquiries can be directed to the corresponding author.
