# Supplementary material for: Digital soft power of cultural heritage: domestic netizens' subjective perception and characteristics of the global influence of traditional festivals
Source: Front Psychol. 2026 Jul 22;17:1896944. doi: 10.3389/fpsyg.2026.1896944 (PMC13439612; doi:10.3389/fpsyg.2026.1896944)
Supplement: Supplementary file 1 [file Data_Sheet_1.zip › DataSheet_2_Codebook.docx]

**Questionnaire: Digital Soft Power of Cultural Heritage: Perceived Measurement and Characteristics of the Global Influence of Traditional Festivals**
**测量问卷：文化遗产的数字软实力：传统节日全球影响力的感知测度与特征**

**Informed Consent / 知情同意书**
尊敬的参与者，您好！这是一项关于“数字媒体使用与网络文化感知”的学术调查。完成问卷大约需要 3-5 分钟。您的所有回答将被严格保密，数据进行脱敏处理，且仅供学术研究使用。本研究不包含任何商业目的或身心风险。参与纯属自愿，您可以随时退出。感谢您的支持！
Dear Participant, this is an academic survey regarding “digital media usage and online cultural perceptions.” Completing the survey takes approximately 3-5 minutes. All your responses will be kept strictly confidential, anonymized, and used solely for academic research purposes . This study involves no commercial purposes or physical/psychological risks. Participation is entirely voluntary, and you may withdraw at any time. Thank you for your support!

#### Section 1: Demographics & Ethics / 第一部分：基本信息与伦理确认

**1. [Ethical Consent] I am at least 18 years old, have read the above information, and voluntarily agree to participate in this study. / 【伦理确认】我已满18周岁，已阅读上述信息，并自愿同意参与本研究。**
A. Yes, I agree to participate. / 是的，我同意参与。
B. No / Under 18 years old (Skip to end) / 不同意 / 未满18岁（逻辑跳转：结束问卷）

**2. What is your gender? / 您的性别是：**
A. Male / 男
B. Female / 女
C. Other / Prefer not to say / 其他 / 不愿透露

**3. What is your age group? / 您的年龄段是：**
A. 18 - 25 years old / 18 - 25岁
B. 26 - 35 years old / 26 - 35岁
C. 36 - 45 years old / 36 - 45岁
D. 46 - 55 years old / 46 - 55岁
E. 56 years old and above / 56岁及以上

**4. What is your highest level of education? / 您的受教育程度是：**
A. High school or below / 高中/中专及以下
B. Associate degree / 大专
C. Bachelor’s degree / 本科
D. Master’s degree or above / 硕士研究生及以上

**5. Do you have any experience studying, working, or living overseas for more than 3 consecutive months? / 您是否有在海外（连续3个月以上）学习、工作或生活的经历？**
A. Yes / 是
B. No / 否

#### **Section 2: Digital Media Usage Patterns / 第二部分：数字媒体使用特征**

**6. On average, how much total time do you spend daily on social media platforms (including domestic and overseas platforms)? / 平均而言，您每天在社交媒体（含国内及海外平台）上花费的总时间大约是：**
A. Less than 1 hour / 1小时以内
B. 1 - 3 hours / 1 - 3小时
C. 3 - 5 hours / 3 - 5小时
D. More than 5 hours / 5小时以上

**7. In the past year, how often have you actively read, liked, commented on, or shared content related to “Chinese traditional festivals” on social media? / 过去一年内，您在社交媒体上主动阅读、点赞、评论或转发与“中国传统节日”相关内容的频率是：**
A. Never / 从不
B. Rarely / 很少
C. Occasionally / 偶尔
D. Often / 经常
E. Always / 总是

#### **Section 3: Psychological & Perceptual Measurements / 第三部分：核心心理与感知测度**

(Note: Items 8-10 are measured on a 5-point Likert scale: 1 = Strongly Disagree / 非常不同意, 2 = Somewhat Disagree / 比较不同意, 3 = Neutral / 一般, 4 = Somewhat Agree / 比较同意, 5 = Strongly Agree / 非常同意)

**8. [Digital Modality Tendency / 数字媒体模态倾向] Please rate your level of agreement with the following statements: / 请评估以下陈述与您实际习惯的相符程度：**

1.Compared to plain text, I rely more on short videos or live streams (e.g., TikTok, Douyin) to access the global transmission dynamics of traditional festivals. / 相比于纯文字，我更依赖通过“短视频或直播”来获取有关传统节日的全球传播动态。

2.Audiovisual digital content (e.g., Vlogs, video clips) makes me feel the overseas influence of Chinese traditional culture more directly than text and images. / 我认为视听结合的数字内容（如Vlog、影视剪辑）比图文更能让我直接感受到中国传统文化的海外影响力。

**9. [Perceived Global Influence / 文化全球影响力感知测度] Based on your experience in the digital interconnected world, please rate the following: / 请根据您在数字互联世界中的感受，对以下陈述进行评分：**

1.I often see foreign netizens discussing or even celebrating Chinese traditional festivals on the international internet or social media. / 我能在国际互联网或社交媒体上，经常看到外国网友讨论甚至庆祝中国的传统节日。

2.I believe the cultural symbols of Chinese traditional festivals (e.g., the dragon, red envelopes, mooncakes) hold strong appeal for overseas audiences. / 我认为中国传统节日的文化符号（如春节的龙、红包、中秋的月饼等）对海外受众具有很强的吸引力。

3.**[Attention Check]** To ensure data validity, please be sure to select “Somewhat Disagree” for this item. / **【注意力测试题】** 为了保证数据有效性，请您务必在此题选择“比较不同意”。

4.I feel that overseas audiences increasingly understand and empathize with the values behind Chinese traditional festivals (e.g., family reunion, respect for nature). / 我感觉到海外受众越来越能理解并共情中国传统节日背后的价值观（如家庭团圆、敬重自然等）。

5.Overall, I believe digital media effectively showcases the cultural soft power of Chinese traditional festivals on a global scale. / 总体而言，我认为数字媒体有效展示了中国传统节日在全球范围内的文化软实力。

**10. [Collective Self-Esteem & Cultural Empowerment / 集体自尊与数字文化赋能感] Please rate your true alignment with these statements: / 请评估以下陈述与您内心真实想法的相符程度：**

1.Generally, I feel that the international internet community evaluates my cultural group (Chinese culture) positively. / 总体而言，我觉得国际互联网社会对我的文化群体（中华文化）评价是积极的。

2.In the interconnected global network, I feel deeply proud to see traditional festivals going global. / 在互联世界的网络环境中，看到传统节日出海，我对此感到深深的自豪。(编者注：已剔除原有的“跨文化”字眼)

3.I firmly believe that ordinary netizens like myself can make small but important contributions to the global spread of Chinese traditional culture through active engagement on social media. / 我确信像我这样的普通网民，通过在社交媒体上的积极互动，也能为中国传统文化的全球传播做出微小但重要的贡献。

4.When I see high-quality content promoting Chinese traditional festivals on social media, my desire to actively spread the culture as a netizen is greatly stimulated. / 当我在社交媒体上看到高质量的中国传统节日传播内容时，我感到自己作为网民的文化传播意愿被极大激发了。
