# Supplementary material for: Digital soft power of cultural heritage: domestic netizens' subjective perception and characteristics of the global influence of traditional festivals
Source: Front Psychol. 2026 Jul 22;17:1896944. doi: 10.3389/fpsyg.2026.1896944 (PMC13439612; doi:10.3389/fpsyg.2026.1896944)
Supplement: Supplementary file 1 [file Data_Sheet_1.zip › DataSheet_1_Questionnaire.docx]

**Data Codebook / 数据编码本**
**Dataset:** Cleaned_Survey_Data_1540.csv

*Note: This codebook provides the variable names, descriptions, and coding schemes used in the final dataset for the study “Digital Soft Power of Cultural Heritage: Perceived Measurement and Characteristics of the Global Influence of Traditional Festivals”*
*说明：本编码本提供了本研究最终使用数据集中涉及的变量名称、描述及计分赋值方案。*

#### **1. Demographic & Control Variables / 基本人口学与控制变量**

| Variable Name (数据列名) | Construct / Description (变量描述) | Coding Scheme / Values (赋值方案) |
| --- | --- | --- |
| Q2_Gender | Gender of the participant / 性别 | 1 = Male (男) 2 = Female (女) |
| Q3_Age | Age group / 年龄段 | 1 = 18-25 2 = 26-35 3 = 36-45 4 = 46-55 5 = 56 and above (56岁及以上) |
| Q4_Edu | Highest education level / 最高学历 | 1 = High school or below (高中/中专及以下) 2 = Associate degree (大专) 3 = Bachelor’s degree (本科) 4 = Master’s degree or above (硕士及以上) |
| Q5_Overseas | Overseas experience (>3 months) / 海外经历(连续3个月以上) | 1 = Yes (有) 2 = No (无) |

#### **2. Main Constructs (Likert Scale Items) / 核心结构变量 (李克特量表题)**

*All items below (except Q6) are measured on a 5-point Likert scale:*
*(1 = Strongly Disagree/非常不同意, 2 = Somewhat Disagree/比较不同意, 3 = Neutral/一般, 4 = Somewhat Agree/比较同意, 5 = Strongly Agree/非常同意)*

| Variable Name (数据列名) | Dimension (所属维度) | Item Description (题目核心描述) |
| --- | --- | --- |
| Q6_DMU1 | Digital Media Usage (数字媒体使用) | Daily social media time / 日均社交媒体时长 *(Note: Coded 1 to 4: 1=<1h, 2=1-3h, 3=3-5h, 4=>5h)* |
| Q7_DMU2 | Digital Media Usage (数字媒体使用) | Frequency of digital interaction with traditional festivals / 传统节日内容互动频率 *(1=Never, 5=Always)* |
| Q8_1_MOD1 | Digital Modality Tendency (数字媒体模态依赖) | Reliance on short videos/live streams over text / 相比图文更依赖短视频/直播 |
| Q8_2_MOD2 | Digital Modality Tendency (数字媒体模态依赖) | Dynamic audiovisuals convey influence more directly / 视听动态更直接传递影响力 |
| Q9_1_INF1 | Perceived Global Influence (全球影响力感知) | Seeing foreign netizens discussing/celebrating festivals / 看到外国网友讨论传统节日 |
| Q9_2_INF2 | Perceived Global Influence (全球影响力感知) | Cultural symbols have strong appeal overseas / 节日文化符号对海外有吸引力 |
| Q9_3_ATTN | Attention Check (注意力检验题) | *Data Quality Control / 质控题* *(Valid cases must equal 2 / 有效样本必须等于 2，即“比较不同意”。Cleaned 数据集中其它值已剔除)* |
| Q9_4_INF3 | Perceived Global Influence (全球影响力感知) | Empathy with festival values by overseas audiences / 海外受众对节日价值观的共情 |
| Q9_5_INF4 | Perceived Global Influence (全球影响力感知) | Digital media showcases cultural soft power effectively / 数字媒体有效展示文化软实力 |
| Q10_1_CSE1 | Collective Self-Esteem (集体自尊) | International internet community evaluates my culture positively / 国际网络对中华文化评价积极 |
| Q10_2_CSE2 | Collective Self-Esteem (集体自尊) | Proud to see traditional festivals going global / 看到传统节日出海感到自豪 |
| Q10_3_EMP1 | Digital Cultural Empowerment (数字文化赋能感) | Netizens can contribute to global cultural spread / 网民能为文化全球传播做贡献 |
| Q10_4_EMP2 | Digital Cultural Empowerment (数字文化赋能感) | Content stimulates desire for active cultural spreading / 优质内容激发文化传播意愿 |
